# Editorial: Immune Profile After Autologous Hematopoietic Stem Cell Transplantation for Autoimmune Diseases: Where Do We Stand?

**DOI:** 10.3389/fimmu.2019.03044

**Published:** 2020-01-09

**Authors:** Kelen Cristina Ribeiro Malmegrim, Antoine Toubert, Dominique Farge, Maria Carolina Oliveira

**Affiliations:** ^1^Department of Clinical Analysis, Toxicology and Food Science, School of Pharmaceutical Sciences of Ribeirão Preto, University of São Paulo, Ribeirão Preto, Brazil; ^2^Center for Cell-Based Therapy, Regional Hemotherapy Center of Ribeirão Preto Medical School, University of São Paulo, Ribeirão Preto, Brazil; ^3^Université de Paris, Institut de Recherche Saint Louis, EMiLy, Paris, France; ^4^INSERM UMRS 1160, Microenvironment, Lymphocyte Development and Homing, Paris, France; ^5^Laboratoire d'Immunologie et d‘Histocompatibilité, AP-HP, Hopital Saint-Louis, Paris, France; ^6^Unité de Médecine Interne, Maladies Auto-immunes et Pathologie Vasculaire (UF 04), Hôpital St-Louis, AP-HP, Paris, France; ^7^Université de Paris, IRSL, Recherche Clinique Appliquée à l'Hématologie, Paris, France; ^8^Department of Medicine, McGill University, Montreal, QC, Canada; ^9^Division of Rheumatology, Allergy, Immunology and Immunotherapy, Department of Internal Medicine, Ribeirão Preto Medical School, University of São Paulo, Ribeirão Preto, Brazil

**Keywords:** hematopoietic stem cell transplantation, autoimmune diseases, immune reconstitution, immune monitoring, cell therapy

Autologous hematopoietic stem cell transplantation (AHSCT) induces long-term remission in autoimmune diseases (AD) without further use of immunosuppression (1, 2). Recently, randomized trials have proven greater efficacy of AHSCT when compared to conventional therapies for multiple and systemic sclerosis (3–6). However, despite the overall positive outcomes, subgroups of patients reactivate the AD after AHSCT due to reasons not yet completely understood, indicating that additional specific immunological interventions may still be required to improve or sustain therapeutic efficacy of AHSCT.

This Frontiers Research Topic, combines reviews, opinions, and original research from the most active researchers in the field of AHSCT for AD. Here, clinical outcomes and immune mechanisms of AHSCT, as well as insights for future studies are presented in the setting of different AD.

Different concepts, from basic knowledge to translational medicine, are integrated in two Perspective articles. Malmegrim et al. critically review the current knowledge about the operating immune mechanisms of AHSCT for AD, and propose strategies for future immune monitoring studies and biobanking. Harris et al. present data from a trial on AHSCT for poor prognosis multiple sclerosis to illustrate post-transplantation immune reconstitution and discuss experimental challenges and strategies to identify biomarkers of clinical response to AHSCT.

Two review articles address mechanistic effects of AHSCT. Massey et al. describe the pathogenesis of multiple sclerosis (MS) and how AHSCT restores immunological balance and, therefore, tolerance. The authors revise most of the available clinical trials on HSCT for MS and discuss aspects such as the lymphopenia-induced proliferation (LIP) that takes place early after transplantation and how it correlates with the later thymic rebound and T-cell diversification. Pockley et al. also describe the dynamics of immune reconstitution that follows AHSCT in AD patients and how it can be extrapolated to Crohn's disease patients. Post-transplantation evidence of immune rejuvenation, with thymic rebound and improvement of regulatory mechanisms are shown in Crohn's disease patients, as well as increase in T-cell receptor (TCR) repertoire diversity in mucosal biopsies. The authors also discuss both established and potential effects of AHSCT on the innate immune system, which plays an important role in inflammatory bowel disease pathogenesis, and how innate cells may contribute to the high rate of Crohn's disease reactivations after transplantation.

Del Papa and Pignataro provide a very detailed and updated review on the mechanisms associated with vascular damage and repair in systemic sclerosis patients. The roles of mature endothelial cells and of endothelial progenitor cells (EPCs) are thoroughly described in the context of a disease characterized by diffuse microvasculopathy and endothelial damage. The authors discuss future EPC-based approaches, using either direct cell transplantation or pharmacological stimuli, aiming to promote endothelial repair. This important overview helps us understand possible pathogenic targets for future strategies involving cell therapy in systemic sclerosis.

Couri et al. and van Megen et al. contribute with their opinions on AHSCT for insulin-dependent diabetes mellitus (T1D). Although very consistent and evidence-supported, the opinions of these two groups differ slightly. The former discuss how AHSCT is able to promote temporary, but meaningful pancreatic beta cell preservation, and thus improve glycemic control. However, since the beneficial effects of AHSCT are transient, more intense immunosuppressive strategies may be warranted in future studies, possibly combined with cell-replacement approaches. On the less full side of the glass, van Megen et al. describe AHSCT as a still controversial issue. Ethical concerns such as transplant-associated risk, long-term toxicity, and enrolment of children are discussed, as well as the lack of substantial evidence for irrefutably beneficial results, despite the appeal of reducing long-term complications of T1D. The authors discuss the available information of immunological analyses before and after AHSCT and suggest a more personalized approach to enroll patients for transplantation.

Autologous HSCT for multiple sclerosis leads to abrogation of new clinical relapses and brain lesions. In parallel, there is selective reduction of Th17, but not Th1, cell population and activity. Darlington et al. demonstrate an increase in the kinetics of natural killer (NK) cell reconstitution, when compared to CD4+ T cells, in MS patients post-AHSCT. The resulting increased NK cell:CD4+ T cell ratio correlated with a decrease in Th17 responses. The authors suggest that rapid reconstitution of NK cells following AHSCT contribute to the suppression of Th17 re-emergence, highlighting the importance of NK cells in the post-transplantation setting.

Ben Nasr et al. report that *ex vivo* modulation of hematopoietic stem and progenitor cells with prostaglandins (PGs) increases their immunoregulatory properties by upregulating expression of the immune checkpoint-signaling molecule PD-L1. When tested in murine and human *in vitro* autoimmune assays, PG-modulated progenitor cells were shown to diminish the autoreactive T cell response. The use of PG-modulated progenitors may thus become an attractive and novel treatment for T1D, thus circumventing immunosuppression-related toxicity.

Two other contributions demonstrate that the programmed death-1 (PD-1) signaling pathway may control autoimmunity. T cells in a lymphopenic environment undergo LIP to fill the available “niche” as defined by (self) peptide:MHC (pMHC) complexes with which the TCRs interact and receive at least a weak “tonic” signal to promote T cell survival. The numbers of cells and diversity of the peripheral T-cell pool are controlled by intra and interclonal competition for resources, which together define T-cell “space.” Ellestad et al. found that PD-1 controls pMHC-dependent tonic signals to T cells, independently of IL-7 signaling, at least when available pMHC is abundant. These data suggest that therapies aimed at reducing TCR signaling during the early phases of T cell reconstitution may be more effective than approaches that aim to limit homeostatic cytokine-mediated signals to T cells.

Finally, Ellestad et al. determine that PD-1 is upregulated on CD4+ T-cells undergoing the natural LIP characteristic of the neonatal period. Newly generated T cells lacking PD-1 maintained an enhanced autoimmune potential even after residence in a lymphoreplete periphery, emphasizing the importance of PD-1 in the establishment of peripheral tolerance. Neither Fas nor perforin-dependent killing mechanisms were required for autoimmunity, while host MHC-II expression was critical, suggesting that LIP-driven autoimmunity in the absence of PD-1 may primarily result from a CD4+ T-cell-mediated systemic cytokinemia. Their data suggest that even in a lymphoreplete adult host, peripheral newly generated T cells retain a potential for LIP-driven autoimmunity in the absence of PD-1.

Collectively, the articles from this Research Topic contribute to increase the knowledge of the field. Important aspects about the modulation of the immune system in autoimmune diseases are discussed, from cellular to more molecular approaches, and from bedside to bench, which in the future should reverse back to the bedside.

## Author Contributions

KM and MO wrote the initial draft of the editorial. AT and DF revised and approved the manuscript.

### Conflict of Interest

The authors declare that the research was conducted in the absence of any commercial or financial relationships that could be construed as a potential conflict of interest.
